# A conceptual model of mindful organizing for effective safety and crisis management. The role of organizational culture

**DOI:** 10.1007/s12144-022-03702-x

**Published:** 2022-09-05

**Authors:** Laura Petitta, Mario Martínez-Córcoles

**Affiliations:** 1 Department of Psychology, Sapienza University of Rome, Via dei Marsi, 78, 00185 Rome, Italy; 2 Research Institute On Personnel Psychology, Organizational Development, and Quality of Working Life (IDOCAL), University of Valencia, Valencia, Spain

**Keywords:** Mindful Organizing, Organizational culture, Risk management, Crisis management, Attraction-selection-attrition, Safety

## Abstract

The Covid-19 pandemic has involved nations world-wide in the necessity to manage and control the spread of infection, and challenged organizations to effectively counteract an unchartered medical crisis while preserving the safety of workers. While the pandemic and geopolitical turmoil caused by the war in Ukraine are recent examples of complex environments that require effective safety and crisis management, organizations may generally need to find ways to deal with the unexpected and reliably perform in the face of fluctuations. Mindful organizing (MO) is defined as the collective capability to detect discriminatory details about emerging issues and act swiftly in response to these details, thus allowing members to anticipate, and recover from, any errors or unexpected events that arise. Organizational culture refers to the mindset shared among members which orients their actions and thus qualifies as a relevant contextual factor that determines whether the specific forms of perceiving and acting entailed by MO may emerge in an organization. The present paper aimed to propose a conceptual model linking organizational culture, MO and organizational outcomes (i.e., safety, reliability, crisis management), and delineate arguments to address the match/mismatch between MO and culture types. Specifically, it is proposed that organizational culture determines the way an organization develops MO and the subsequent ability to handle unexpected events which might jeopardize organizational effectiveness and safety. Our contribution bridges the still disparate fields of MO and organizational culture, and provides scholars and practitioners with a complexity- and uncertainty-sensitive integrative framework in order to intervene on organizational outcomes.

The Covid-19 pandemic has involved all nations world-wide in the necessity to manage and control the spread of infection, and has brought to the forefront the challenge for organizations to effectively counteract an unchartered and rapidly escalating medical crisis while preserving the safety and well-being of workers. Initial evidence suggests that organizations distinguished by their commitment to their workforce's health, safety and well-being outperform in the marketplace (Fabius & Phares, 2021), thus confirming how the unprecedented challenges of combatting the pandemic have tightly intertwined crisis management (i.e., skills to cope with disruptive unexpected events after they have occurred; Bundy et al., 2017*)* and risk management (i.e., skills to cope with threats before they occur; Amalberti, 2013) associated with Covid-19 hazards. Similarly, the geopolitical turmoil caused by the war in Ukraine is an additional striking example of how organizations are increasingly called to face nowadays environments made unpredictable and unstable by unexpected global events, and to cope effectively with the unforeseeable. While the pandemic and the geopolitical consequences of the war in Ukraine are just recent and striking examples of uncertain and complex environments that require effective health and safety crisis management, effectively dealing with the unexpected and uncertainties has increasingly and more generally become the key for organizations survival and success. In such uncertain and complex environments, organizations can achieve highly safe and reliable performance by organizing mindfully, that is, by continuously developing workers sensitivity to the unexpected and capability to detect weak signals regarding emerging issues, which ultimately enables the system to effectively manage fluctuations (Roe & Schulman, 2008; Schulman, 1993; Sutcliffe, 2018). Literature suggests that organizational mindfulness (i.e., a collective state of alertness and active awareness characterized by heightened attention, openness to new information, and awareness of multiple perspectives; Langer, 1989; Ryle, 1949; Sternberg, 2000) enables organizational members to react more readily to problems that occur in ongoing operations and respond to their external environments more effectively (Weick & Sutcliffe, 2001), thus qualifying as a key factor in determining organizational effectiveness rooted into its ability to manage crises and perform safely and stably in the face of uncertainties and fluctuations (i.e., reliability).

Mindful organizing refers to the collective capability to detect discriminatory details associated with emerging issues and act swiftly in response to these details (Weick et al., 1999). Similar to the concept of mindfulness (i.e., awareness of the present moment; Kabat-Zinn, 1990), mindful organizing refers to a set of organizational processes collectively enacted by all members that focus attention on weak signals that may pose a threat to organizational operations, thus cultivating enriched awareness that enables the discovery and correction of unexpected events capable of escalation. Initially developed mainly in relation to the management of safety, mindful organizing represents a form of crisis management and is therefore potentially applicable to any circumstance and business field wherein organizations have to deal with unexpected and disruptive surprises. While mindful organizing is potentially applicable to all organizations aiming to achieve effective and reliable performance (Martínez-Córcoles & Vogus, 2020), the role of contextual factors such as organizational culture (i.e., shared values and patterns of behaviors that orient members’ action; Schein, 1985) in determining whether mindful organizing may emerge and/or become institutionalized throughout an organization is still unexplored. Indeed, Weick and Sutcliffe (2007) suggest that in order to effectively manage the unexpected, real mindfulness in any organization may require “changing the organizational culture”. Importantly, while the literature (Weick & Sutcliffe, 2007) explicitly points to organizational culture change in order to build and develop mindfulness in any organization beyond HROs, it does not address the theoretical foundations for this link and the way to cover the gap between the actual culture of an organization and the desirable culture that effectively underpins and makes operative mindful organizing processes. Noteworthy, a study on organizations coping with early pandemic spreading in the US suggests that workplace practices and cultures that adopted COVID-19 prevention measures resulted in more responsible worker safety behavior both in work and non-work situations, thus contributing to combat the outbreak even at the larger community level (Probst et al., 2021). Moreover, at the global level, the literature suggests that countries act in harmony with their local cultural characteristics in the formal or informal practices of their fight against outbreaks (Gokmen et al., 2021), and national culture has determined different levels of success on COVID-19 pandemic management and control (Han et al., 2020).

The aim of the present paper is to propose a conceptual model linking organizational culture, mindful organizing and organizational collective outcomes. Specifically, it is proposed that organizational culture, and safety culture in particular, determines the way organizations develop mindful organizing, and the subsequent ability to handle unexpected and disruptive events that put organizational effectiveness and safety at stake (see Fig. 2). The contribution of our study to the extant literature is at least four-fold. First, we respond to the recent call from researchers in the field of safety and mindful organizing to examine organizational contextual variables as causes of mindful organizing (e.g., Martínez-Córcoles & Vogus, 2020) as well as to address the still overlooked question on how to enhance mindful organizing processes (Klockner, 2018). Our focus on organizational culture as an antecedent of mindful organizing may have relevant theoretical and practical implications by providing conceptual and operational distinctions between the two constructs, and proposing effective means of improving organizational effectiveness. Second, we bridge organizational culture and mindful organizing literatures and their contribution in predicting effective organizational performance and safety, and provide an integrative model that can orient researchers and practitioners to prevent poor safety outcomes and develop crisis and risk management skills. Third, our paper contributes to the clarification and delimitation of both constructs (safety culture and mindful organizing). Despite the fact that some authors have stated that mindful organizing may be related to error management culture (e.g., Vogus, 2011), or just culture (e.g., Callari et al., 2019), the literature linking these two important phenomena is still scarce to our knowledge. We note that at the moment, a blurred line between safety culture and mindful organizing exists, and that a clear delimitation of boundaries is needed. Fourth, we propose a conceptual model with specific details about how to match safety culture with mindful organizing to deal with surprises, unexpected events and unsafe acts, thus preventing workplace accidents and developing crisis management. Organizations using (or willing to use) safety culture/mindful organizing approaches will be provided with an integrative framework which may help diagnosis and intervention on organizational effectiveness by mirroring and addressing the complexities and challenges of reality and its uncertainties.

Below we present the theoretical foundations for mindful organizing. Next, we introduce the theoretical framework for organizational culture, and safety culture in particular, and lay the basis to discuss its relevance for mindful organizing. We then define the main collective outcomes associated with organizational effectiveness and discuss how they connect to mindful organizing. Finally, we provide an overview of the proposed conceptual model linking culture, mindful organizing and organizational outcomes, and delineate arguments to address the matching of mindful organizing processes with organizational culture types.

## Mindful organizing: The key dimensions

Mindful organizing is defined as the collective capability to detect discriminatory details regarding emerging issues and act swiftly in response to these details (Weick et al., 1999). The construct emerged in the late twentieth century, when Weick et al. (1999) extended Langer’s definition of individual mindfulness to a collective level (Sutcliffe, 2018). Langer (1989, 2005) intended mindfulness as a state of alertness and lively awareness of an individual, wherein attention is focused on events occurring in the present moment, both internally and externally. By contrast, mindful organizing is not an intra-psychic process that occurs in the minds of individuals (Morgeson & Hofmann, 1999) but rather, a set of organizational processes and practices enacted by organizational members that focus attention on salient stimuli that may pose a threat to the operation of the organization, leading to corrective action (Vogus & Sutcliffe, 2012). As such, mindful organizing is a bottom-up phenomenon directly related to the behaviors carried out by organizational members and, more importantly, is closely related to the repertoire of action capabilities of an organizational system. Specifically, enriched awareness of reality may heighten members’ attention to weak signals that surface out of the continuous stream of events that flow through daily activities and have the potential for error or catastrophes, thus enabling both the discovery and correction of unexpected events capable of escalation. As such, in mindful organizing the quality of attention and the enriched awareness at the collective organizational level is also linked to what people decide to do with what they notice and to the strategies they develop on alternative ways to deal with the abnormal (Weick et al., 1999). From a workplace perspective, mindfulness proved to be a positive characteristic that enables individuals to respond to their external environments more effectively and thus empowers operations that occur in organizations (Weick & Sutcliffe, 2001). Consistently, empirical research has demonstrated that mindfulness in organizations affects organizational outcomes at both the individual and organizational levels. For example, mindful organizing positively predicts higher alertness and attention to weak signals (Rerup, 2009), negatively affects medication errors (Ausserhofer et al., 2013; Vogus & Sutcliffe, 2007a, b) and patient falls (Vogus & Sutcliffe, 2007a), positively predicts safety compliance (Gracia et al., 2020) and job satisfaction (Renecle et al., 2020) and determines higher creativity (Runco, 2007; Runco & Albert, 1990), innovation and learning (Levinthal & Rerup, 2006). Overall, mindful organizing associates to higher organizational attention (Weick & Sutcliffe, 2006), security (Butler & Gray, 2006), as well as adaptation and in-role and extra-role performance (Renecle et al., 2021; Rerup, 2005; Senge et al., 2005; Weick & Sutcliffe, 2007).

While research on the outcomes of mindful organizing has flourished driven by the efforts to prove its benefits for organizational life, literature has been criticized for having poorly understood and narrowly considered its antecedents (Argote, 2006; Gracia et al., 2020) and, to date, there is still limited understanding of how mindful organizing emerges, spreads, and becomes institutionalized throughout an organization (Martínez-Córcoles & Vogus, 2020). Among the main antecedents of mindful organizing, literature suggests that individual characteristics such as knowledge and abilities (Roe & Schulman, 2008) make mindful organizing possible. At the group-level, leadership (Sutcliffe et al., 2016) and team safety climate (Renecle et al., 2020, 2021) are potential predictors of mindful organizing. Organizational-level antecedents seem to be the most studied factors and literature points to reliability-enhancing work practices (REWPs) such as selective staffing, extensive training, developmental performance appraisal, and decentralized decision-making (Vogus & Iacobucci, 2016), trust and respect (Vogus & Iacobucci, 2016), and human resource and work design practices (Vogus & Welbourne, 2003). Finally, Weick and Sutcliffe (2007) proposed three main antecedents of organizational mindfulness: awareness of potential problems (e.g., collective concern for mis-understanding of things), tendency toward carelessness (e.g., collective stereotyped way of thinking), and complexity of the organization (e.g., numbers of units and their interactivity). Noteworthy, while the latter is related to structural variables of an organizational system, the first two aspects pertain to culture-related factors such as shared values and behavioral schemas (Schein, 1985).

**Fig. 1:**
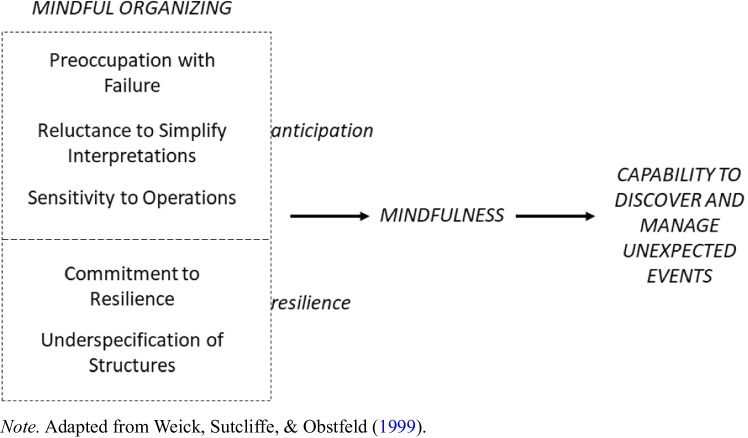
The link between mindful organizing, mindfulness and unexpected events. *Note.* Adapted from Weick et al. (1999)

In the theoretical framework proposed by Weick et al. (1999), mindful organizing entails five different and separate processes that jointly interrelate to induce a rich state of collective attention and awareness of details (i.e., mindfulness) which, in turn, facilitates the detection and correction of unexpected events capable of escalation (see Fig. 1). The five interrelated dimensions of mindful organizing are (Weick et al., 1999): a) preoccupation with failure, b) reluctance to simplify interpretations, c) sensitivity to operations, d) commitment to resilience and e) deference to expertise. Noteworthy, the first three processes of mindful organizing involve *anticipation,* or the collective capability to anticipate unexpected problems, whereas the last two processes have to do with *resilience,* or the collective capacity to contain unexpected events once they arise (Weick & Sutcliffe, 2007). Specifically, while anticipation refers to the prediction and prevention of potential problems before damage is done, resilience is the capacity to cope, in the moment, with unexpected problems after they have become manifest and learn to bounce back and recover in the future (Wildavski, 1991). These two clusters reflect Weick’s et al. (1999) conceptualization of mindful organizing understood as both enriched awareness of ongoing experiences as well as the capacity for action and copying with events. Below we describe each dimension in details.

*Preoccupation with failure* refers to the ongoing organizational awareness of the possibility of system failure and the consideration of any failure or near miss to be an indicator of potentially larger problems (LaPorte & Consolini, 1991). This dimension of mindful organizing brings to the forefront the relevance of paying attention not only to errors but also to near misses understood as weak signals of potential malfunctions of some portion of the organization. As such, failure is not necessarily lack of success but refers to any “dysfunctional response” to success. Therefore, even when the organization is used to smooth functioning and success, preoccupation with failure lay the ground for continuous organizational improvement by warning against the consequences of success like inertia, reduced attention and expectation of automatic success in the future (Weick et al., 1999). In order to increase attentiveness to failures, workers must first recognize the potential of technological systems for surprises and be aware of system interdependencies, causal chains and sequence of events. Rather than the “waiting to happen” approach, workers try to expand their attention proactively toward the “possible to happen”, being even suspicious about success. By actively searching for and attending to seemingly insignificant weak signals, people can more quickly detect if the system is acting in unexpected ways (Sutcliffe, 2018). Examples of behaviors oriented to foster preoccupation with failures are management and supervisor support (e.g., creating a safe psychological climate), broadening the variety of data points available for learning and gathering extra-data from failures and encouraging and rewarding reporting behaviors. For example, maintenance workers come into contact with the largest numbers of failures or near-misses, thus having an ongoing sense of vulnerability and potential problems at earlier stages of development. Organizations that focus detailed attention to these observations and create internal connections among different parts of the system may enlarge their database for learning (Weick et al., 1999).

*Reluctance to simplify interpretations* puts under the spotlight how organizational members handle complex tasks and their temptation to simplifying the manner in which the current situation is interpreted. Simplifications are worldviews or mindsets that drive members to ignore data and keep going, thus allowing anomalies to accumulate and undesired consequences to grow more serious. Ultimately, simplifications increase the likelihood of eventual surprise (Turner, 1978). On the contrary, reluctance to simplify interpretations refers to the organizational attitude to constantly assess whether the diagnosis of the present and likely future situation is accurate enough to achieve organizational goals without encountering unexpected difficulties that lead to catastrophe. As such, the challenge is to find a balance between which aspects of the current set of problems facing an organization are prudent to ignore and which should be attended to. While organizations generally tend to overlook the question of what they ignore, engaging in reluctance to simplify interpretation means valuing this question and actively exploring what members do not know. Reluctance to simplification is a process achieved by two different exercises: divergence and integration of views. In order to avoid mental simplifications of the functioning of a system, organizations must resist workers’ tendency to generate simple cause-effects assumptions, and seek divergent perspectives to expand the current set of assumptions. However, an exercise of integration is needed so that heterogeneous views about the same operational aspects may be transformed into a nuanced picture of ongoing operations. This exercise of integration consists of cooperation efforts in re-negotiating and integrating divergent views (Weick et al., 1999). According to Sutcliffe (2018), this integration of different approaches is easier to attain when a strong relational foundation is based on respect and trust exists. Both simplification and reluctance to simplify interpretations are ways to view the world and its complexities that could be cultivated by organizations through socialization. In particular, reluctance to simplify could be developed by socializing people to notice more and cultivating requisite variety (Weick et al., 1999).

*Sensitivity to operations* refers to members’ ability to construct and, more importantly, maintain an integrated big picture of operations in the moment, a cognitive map that allows them to integrate diverse inputs and be receptive to any alteration or problem during ongoing operations (Weick et al., 1999). Being in close touch with what is happening here and now allows members to catch small problems in real-time before they become bigger. Sensitivity to operations is similar to the notion of situational awareness and is reflected in descriptive words such as struggle for alertness, misinterpretation, overload, distraction, mixed signals, surprise, vigilance, near misses, warnings and anomalies, which all portray the concern to catch errors in the moment and dangers inherent in the loss of this sensitivity (Miller & Woods, 1997). Maintaining a broad operational awareness is particularly challenging for members under situations of production pressure and overload; yet, organizations with high sensitivity to operations tend to be more self-conscious in dealing with pressures of overload and its effects on judgment and performance, thus exhibiting extraordinary attentiveness to the incipient overloading of any one of its members (Reason, 1990). Increased sensitivity to operations is created when organizational members carry out collective actions based on information sharing and create an up-to date understanding of the distributed tasks and expertise, so that these are appropriately utilized in the face of ongoing operations (Vogus, 2011). Examples of these collective (i.e., cross-level and cross-disciplinary) actions are continuous formulation of scenarios, monitoring operations and shared story building (Weick et al., 1999). By enlarging a shared understanding of the whole system, organizations can foresee possible scenarios and make small ongoing adjustments to prevent incubation of latent failures (Sutcliffe, 2018) and eventually, undesired outcomes. Overall, “it is collective knowledge of failures, details, potentials for recovery, and relevant past experience, gathered into mindful processing, that provides the context within which present operations either make sense or are reconstructed to make sense” (Weick et al., 1999, p. 45).

To summarize, preoccupation with failure, reluctance to simplify interpretations and sensitivity to operations jointly enable a richer representation of the complexity of operations, and provide a greater collective ability to discern discriminatory details about ongoing emerging issues (e.g., uncovering blind spots and threats). Therefore, they are crucial to *anticipate* vulnerabilities or contingencies and prevent them from accumulating or incubating into bigger problems (Sutcliffe, 2018).

*Commitment to resilience* involves developing members’ capabilities to handle unanticipated events by improvising and adapting to contingencies (Van Dyck et al., 2005). Noteworthy, resilience is considered both the ability to cope with surprises in the moment as well as bounce back from errors. It is important to retain both connotations of resilience in order to clearly point out that effective organizations are able not only to absorb change and still persist but also to utilize the change that is absorbed (Wildavski, 1991). Being committed to resilience means to enlarge organizational capabilities to improvise, learn from and adapt to surprises (Sutcliffe & Vogus, 2003). In the preparation for coping with surprises the first step is to embrace them, by accepting and acknowledging the complexity and tightly-coupling nature of the operation, and therefore, its potential to fail. Improvisation is seen as a necessary ability to respond in real-time to maintain the task in question in spite of a turbulent environment. This dimension also entails the capacity to continuously learn from actions (and reactions), integrating them into new and enlarged action repertoires (possible solutions that can be applied). Those organizations with a strong commitment to improve overall capability are characterized by continual training and simulation, varied job experiences, learning from feedback, and rapid ad hoc reconfiguration of networks that facilitate a rapid pooling of cognitive knowledge to cope with uncertainty and imperfect knowledge (Sutcliffe, 2018; Weick et al., 1999). An example of how organizations commit to resilience is their capability to contain emerging crises through informal epistemic networks, understood as informal groups of knowledgeable people that self-organize into ad hoc networks to provide expert problem solving when events get outside of normal operational boundaries (Rochlin, 1989). These informal latent networks represent a strategy for flexible crisis intervention that enables systems to deal with the irreducible and allow for rapid pooling of cognitive knowledge to handle events that were impossible to anticipate and dissolve as soon as normalcy returns. Additionally, organizations committed to resilience also put into place formal initiatives such as support for improvisation. This is because organization’s ability to act on hazards is rooted in their sensitivity to detect those hazards and think about them (Bourrier, 1996).

*Deference to expertise* occurs when decisions migrate to those with the greatest expertise in the problem at hand, regardless of their formal rank (Roberts et al., 1994). Most organizations are orderly systems with clear routines and procedures that define organizational members’ role and orient them in their tasks. Orderly structures are characterized by defining who gains access to what and who makes which decision according to hierarchical rank. However, routines and procedures can act as amplifiers of errors if the structure remains rigid without the capacity to be flexible when unexpected problems arise. A simultaneous combination of orderliness and flexibility requires open access to authority and decision making when needed (losing the filters on who gains access to that), and allowing members to make decisions (Weick et al., 1999). The result is that when there is a need for quick decision making, expertise trumps hierarchical rank, and therefore, a wider range of problems can match a wider range of capabilities (Sutcliffe, 2018). Effective organizations loosen the designation of who is the “important” decision maker, thus allowing decision making to migrate along with problems (Lekka, 2011). Specifically, a mindful flow of activities would be as follows: a) someone notices an anomaly, b) they turn to others in an effort to understand what it means and spread the troublesome cues around, c) they turn to anyone who may contribute with their expertise, thus exposing the cues to a more varied set of capabilities in an effort to diagnose and make sense of the situation, d) a subtle loosening of hierarchy in favor of expertise allows this type of consultation, and e) when hierarchy filters are loosened, people are allowed to pay more attention to inputs and processes are more influenced by temporal connections, thus shaping the structuring of operations on problem-solving rather than high ranking decision making (Weick et al., 1999). According to Weick et al. (1999; p. 49), the “agency” that triggers this loosening is not an edict from the top, but rather a collective, cultural belief that the necessary capabilities lie somewhere in the system and that migrating problems will find them”. As such, invariant mindfulness enables members to detect anomalies as well as the structural constraints (e.g., hierarchy) that make it difficult to comprehend the sense of irregularities and whether they signify a problem or a transient event.

To summarize, commitment to resilience and deference to expertise are the factors that jointly contribute to contain unexpected events once they arise (Weick & Sutcliffe, 2007). The impossibility to fully reduce uncertainty and anticipate all situations and conditions, makes it necessary to not only pay attention to error-prevention (anticipation) but to also focus on error-containment as well (resilience).

### The relevance of culture for mindful organizing

Organizational culture helps individuals make sense of their work world and represents a core group of shared set of assumptions, norms and patterns of behavior which orients organizational action (Schein, 1985). Schein’s (1999) conceptualization of organizational culture incorporates three embedded levels of culture expression, ranging from the most visible layer of the artifacts to the intangible layer of values and norms up to and including the deepest layer of basic assumptions which are both invisible and unconscious. While all three layers tie together into a coherent whole which ultimately defines the specific “gestalt” of the culture of any given organization, each layer also displays specific features. For any organization willing to become aware (i.e., diagnose) and intervene on their culture it is fundamental to master the specific manifestations of each cultural layer as well as their conjoint effect in creating consistent and predictable views across all members. Specifically, the most external and tangible layer refers to organizational artifacts (e.g., language, furniture, dress codes, behaviors), which represent the objective and concrete manifestations of culture. Artifacts are the indicators of organizational culture that are directly observable. The next underlying level includes the organizational norms and values that contribute to shape how artifacts are modelled and manifested. Both norms and values could be explicitly and officially stated by the organization as well as tacitly circulating among its members and enacted as a matter of fact without formal framing. While norms and rules are the landmarks that point members towards the expected standards of any organizational context, values are the relative importance that members place on any item of their working reality and contribute to orient them in investing their energies in what really counts in the situation. The third and deepest cultural level refers to the basic assumptions that members hold about their organizational reality and its functioning, and comes from norms and values automatically enacted on a daily basis which gradually drop out of awareness and come to be taken for granted. Not surprisingly, this constitutes the most stable part of organizational culture and the one most resistant to change. We note that these deep convictions are so ingrained in members’ mind to the point of becoming the trigger of their behaviour without them being aware. Since mindful organizing is in itself constant awareness of reality and its cues, this latter layer of basic assumptions is the most challenging yet most relevant to describe and diagnose in order to practice mindful organizing.

A way to analyze organizational cultures is to find a correspondence between the cultural clues present in a given organization and some predefined categories, which therefore make it possible to place a given cultural configuration in a specific cultural typology. Starting from the assumption that organizational action has a precise internal consistency and logic, more or less easily identifiable, understanding the context can take place by linking information on the functioning of its parts with its social and structural form (Petitta et al., 2017a, b; Schein, 1985). Following this approach, the diagnosis of culture is achieved by finding the correspondence between the cultural clues that appear in a context and the aspects considered emblematic of a specific type of culture (type = model), i.e., a category, or class, in which the organization is made to fit into the cultural characteristics it presents.

In the current paper, we build on Enriquez’s (1970) identification of a typology of organizational culture as a theoretical framework to propose our conceptual model of cultural underpinnings of mindful organizing and organizational outcomes. Specifically, the organizational culture typology proposed by the author can represent a valid reading key for the decoding of the organizational context in relation to its prevailing models and patterns of collective action. The five culture types are *autocratic, bureaucratic, clan-patronage, technocratic* and *cooperative.* The reason for proposing Enriquez's cultural typology is both theoretical and practical in nature. From a theoretical standpoint, a recent review of cultural typologies by Sarki et al. (2017) showed that research in the research area of cultural typologies different researchers have analyzed organizational culture and offered at least fifteen different typologies. Overall, the literature proposals range from Cameron and Freeman’s (1991) culture types (Clan culture, Adhocracy culture, Hierarchy culture, Market culture), up to and including Herminingsih (2014) types (Reward culture, Stability culture, Competitive culture, Performance orientation culture, cooperate social responsibility culture, Innovative culture, Supportive culture), as well as Sarki and Adulhamid (2016) dimensions (Norm of Work Culture, Task Culture, Constructive cultures, Passive/defensive cultures, Bureaucratic Culture, Innovative Culture, Supportive Culture). Consistent with previous comparative analysis of cultural typologies (Machado & Carvalho, 2008) suggesting a recurring incidence of certain themes across different culture types models, the five dimensions of Enriquez’s cultural typology allow to a) capture the main dimensions recurring in the extensively reviewed literature, b) propose the culture type dimensions that suit the concept of category or rather, a class/cluster of characteristics that are class-specific and nonoverlapping with other classes/clusters, and c) propose a number of culture types dimensions in an attempt to provide a comprehensive coverage of most of the studied culture types that are non-overlapping with each other and defined by unique features. From a practical standpoint, the literature (Cameron & Freeman, 1991) suggests that culture types have an important relationship with organizational effectiveness as well as with other organizational attributes, thus providing culture types frameworks for preventive intervention on unwanted safety-related outcomes.

To allow the organization to be placed in one typology, rather than another, Enriquez (1970) mainly reports four parameters that take on specific and different characteristics in each of the five types: the prevailing value, career criteria, communication methods and relational patterns, and the individual needs that are met in the psychological contract with the organization (Rousseau, 1996). For example, organizations willing to assess their cultural identity along their prevailing value may identify which value is more widespread (more typical) in their context: Obedience? Compliance with the rules? Affiliation to privileged groups? Meritocracy? Or the collegiality in the management of the organization? Each of them corresponds to, and is the specific indicator of, the five different culture types.

The *autocratic* culture is characterized by supremacy of authority and imposed leader and by descending communications. The dialogue is reduced to the delivery of directives to be followed and feedback does not exist or is a corrective intervention that emphasizes only the errors to be avoided. Authority is inhibitory and controlling because man is conceived as incapable of self-discipline, subordination is desired and rewarded with professional recognition and career advancement and identification with the authority favors imitative behavior. However, the cultural system remains in equilibrium and also tends to attract individuals compatible with these assets. Specifically, there is a dynamic of deep needs that are mutually satisfied wherein the boss is gratified by the exercise of power and control, while the collaborators are relieved of responsibility and can satisfy a possible need to feel protected (need for protection) and affiliation (McClelland, 1987).

In addition to the autocratic culture, Enriquez (1970) outlines a *bureaucratic* type of culture in which the fundamental value is the norm, and the observance of regulations permeate both the organization of work, hierarchy and career progress. People are required to respect the role boundaries and the standard execution of the foreseen tasks, without particular initiatives. The context is shaped by management action focused on the efficiency of the knowledge of formal procedures. The main objective is to ensure that the person correctly performs the activities foreseen by the role, without particular actual involvement (although in some cases apparent) in the performance of the work. People tend to simply fulfill a task and comply to work requirements, and perform exclusively what is under their competence. Interpersonal relationships are usually formal, or otherwise bound by rules, rather than left to people's spontaneity. The presence of equal rules for all tends to create homologating conditions. Noteworthy, this satisfies people’s security needs linked to the predictability of a context so explicitly normative and standardized, which allows people to count on reassuring landmarks provided by rules and norms.

In a *clan-patronage* culture, it is fundamental to belong to a group in which exchanges of reciprocal benefits are activated between members and leader in the interest of all, and where the paternalistic component is expressed with the superiority and control of the head who dispenses favors and privileges to the members of a group. In turn, people reciprocate the leader with loyal support to him/her and to the whole system of advantageous relationships that are structured in this lobby. Noteworthy, this clientelism revolves around leading people regardless of their formal role and who might have informal power. The main indicator of these cultural convictions is the clear distinctions between those who are inside and those who are outside a given group, and the feeling that alongside a dimension of formal life, a hidden and informal world runs parallel, powerfully active and well-functioning. Moreover, the in-group/out-group logic is intertwined with favoritism, clientelism and privilege. In-group people talk in a spontaneous, informal and jargon way among themselves, while maintaining an attitude of a different kind, and more suited to strangers, with those who are outside the group. In this context the main value is belonginess but to privilege groups. Management initiatives are often a pretext to communicate the privilege of being included or not included in situations that are desirable, beyond the objective of formally declared work. In this culture the dynamics of mutually satisfied needs allow those who have the power and dispense benefits (paternalistic element of culture), to feel important and influential; while to those who receive benefits, feel privileged, protected and safe, also thanks to the group dimension to which they belong and to which they contribute by activating themselves to reciprocate what they have received and to maintain the privileges they have reached (clientelar element), thus contributing to incrementally strengthen the power of authority.

A *technocratic* culture can be characteristic of contexts that produce market-oriented services and goods, and gives value to competence and initiative for achievement and organizational success. Excellent performance and efficiency characterize continuous professional development, with a particular confidence in rationality and goal-orientation to be achieved by appealing to all personal resources. Consistently, management is more likely to focus on investing in knowledge and enhancing professional skills, with the most personal resources and components for successful work being brought into play. Given the organizational stress on achievement, competition is a likely ingredient (either against other workers, or other groups, or against external competitors). The value attributed by the organization to success, and to concrete results, offers individual members the opportunity to satisfy their own need for success and personal fulfillment through work.

Finally, in the *cooperative* culture, value is given to the consent and participation of all members (or their representatives) in decisions. The assumption of autonomy of action is the empowerment of the individual and the fact that everyone responds to their own results, without disengagement or withdrawals of individual contributions which might be dangerous for the overall balance of the organization. In situations of real mutual trust and unconditional involvement of all members like this one, quite rare, management is conducted in a fair way, as much as possible shared and agreed, and is aimed at the common well-being and improvement of every component of the system. The needs that are satisfied are essentially those of affiliation and protection linked to others’ acceptance and to reassuring interpersonal relationship style rooted in conflict prevention through participation.

Overall, consistent with the general notion of typology, the typology presented here encompasses several types of culture, each of them with unique features which do not overlap with the other types (i.e., mutually exclusive) (Marradi, 1990). Even though all types encompassed in the typology could be relevant to explain a determined phenomenon, each one shows its own specific features. As such, when it comes to deciphering the culture of an organization, the diagnosis may show that different culture types coexist, although one can prevail over the others (Valsiner & Connolly, 2002). Specifically, it might be difficult to find a pure form of culture type in an organization, especially if the size is large, thus increasing the likelihood that (a) different departments or organizational units within the organization follow different rules and (b) that alongside the core of most recurrent organizational behaviors, namely the dominant culture, secondary cultural sub-sets coexist (Janson, 1994).

### Organizational safety culture

Workplace safety refers to the policies and procedures in place to ensure the safety and health of employees within a workplace (OSHA, 2016). Therefore, a safety culture model grounded in Enriquez’s (1970) typology of organizational culture conceptualizes the extent to which each culture type (e.g., autocratic, bureaucratic, clan-patronage, technocratic and cooperative) shows different safety-related features, norms and behaviors shared among organizational members (Petitta et al., 2017a). In particular, in an *autocratic* safety culture, superiors (i.e., authority) are the source of safety instructions and directions for workers. Controlling employees is the main strategy to prevent them from making mistakes. Dialogue mainly consists of the one-way and top-down delivery of safety feedback and directives involve corrections which highlight errors to avoid in upcoming situations. In a *bureaucratic* safety culture, the fundamental value is strict rule compliance. Each member strictly adheres to safety rules and regulations set by top level bureaucratic officials (i.e., top management, agencies, local governments). Workers are expected to comply with their role prescription and follow safety procedures. Bureaucratic safety cultures are characterized by high levels of standardization and procedures that uniform and homogenize individual contributions. Within the *clan-patronage* safety culture, powerful people within a group of privileged members provide benefits that individuals seek to obtain and that are available to reciprocate. Because only members of the inner circle can access privileges, this dynamic contributes to creating a context that works with different rules depending on the in-group or out-group membership. Moreover, this in-group/out-group dynamic is only rooted in the instrumental dynamics of personal benefits and convenience rather than belonginess and affection. This is a “two-faced” context that provides members with different safety worldviews and norms dependent upon their current interaction with members of their inner circle versus external individuals. The *technocratic* safety culture is characteristic of contexts that emphasize results, prioritize achievement and therefore, often imply competition to succeed. However, prioritizing production achievement may result in safety violations if competition gets carried away or shortcuts to excellence are engaged by hiding errors, skipping safety steps, etc. Finally, within a *cooperative* safety culture**,** shared management of routines and participative leadership promote collaborative dynamics fostering daily participation in safety issues and outcomes and achieving members consensus.

### Unexpected events, risk and risky behavior

Crisis management refers to ongoing skills and techniques displayed to identify, assess, understand and cope with disruptive and unexpected events that threaten to harm the organization or its stakeholders (Bundy et al., 2017*)*. While crisis management involves dealing with surprises and serious situations *before, during* and *after* they have occurred, risk management refers to assessing potential threats and finding the best ways to avoid those threats *before* they unfold. According to previous literature (e.g., Hollnagel, 1993; Reason, 2008; Roe & Schulman, 2008; Weick & Sutcliffe, 2007), the key concepts related to successful crisis management and organizational outcomes of mindful organizing are: management of unexpected events, reliability, risk and risky behaviors. Below we address each of the concepts separately as well as their intercorrelations where applicable.

The main outcome of mindful organizing is the organization’s ability to manage unexpected events, not just occasionally, but repeatedly. In turn, this organizational ability to manage the unpredictable lays the basis to develop later organizational reliability, defined as the capacity to produce collective outcomes of a certain minimum quality on an ongoing basis (Hannan & Freeman, 1984, p. 153). While reliability is commonly thought to be achieved through the development of highly standardized and invariant routines (Hannan & Freeman, 1984), mindful organizing emphasizes that for a system to remain reliable, it must somehow handle unforeseen situations in ways that forestall unintended consequences. Specifically, unvarying procedures cannot handle what they did not anticipate. As such, rather than relying on repeatability of action (i.e., procedures) as the primary defining quality of organizational reliability, mindful organizing calls into action the relevance of constantly applying processes of cognition (i.e., the five mindful organizing dimensions) directed at flexibly varying and adjusting work processes that uncover and correct unintended consequences. In so doing, the world and contingencies are conceptualized as potentially unknowable and unpredictable; yet, organizations that engage action by using the five cognitive processes of mindful organizing can perform an ongoing mutual re-adjustment to situations and an adaptive activity that generates potential information about capability, vulnerability and the environment (e.g., Landau & Chisholm, 1995). In other words, while procedures should be constantly put to test and revised if necessary, what should remain constant and stable is people’s use of mindful processing (i.e., preoccupation with failure, reluctance to simplify interpretations, sensitivity to operations, commitment to resilience and deference to expertise), which helps detect weak signals such as faults in machinery, substandard materials, or declining compliance, and avoids letting such situational oversights lead to unintended consequences for safety and performance. Overall, the information is lost unless there is continuous mindful awareness of these variations (Weick et al., 1999).

In addition to the discovery and correction of surprises and errors that are capable of escalation and proximal antecedents of poor safety outcomes such as accidents and injuries (Nahrgang et al., 2011), effective organizations also have to deal with risks and hazards, which similarly have the potential to compromise safety. A hazard is defined as something that can cause harm (e.g., electricity, chemicals, working up a ladder, noise, a keyboard, stress). Similar to what has been suggested for unexpected events, mindful organizing proposes that the key to organizational effectiveness is the close relationship between employee mindfulness and their action repertoire. Specifically, organizations that are willing to act on specific hazards are also organizations that are willing to notice those hazards, think about them and enlarge the range of clues they can notice in a mindful manner rather than letting errors accumulate unnoticed because “useless” observations of those hazards are ignored or denied (Weick et al., 1999).

A risk is the chance, high or low, that any hazard will actually cause unwanted consequences or somebody harm. Moreover, a risk has both an objective side (i.e., the probability of an occurrence) as well as a subjective component dependent on the person (e.g., the person's perception of the likelihood of an event or the enactment of risky behaviors). An additional subjective component of risk is risky behavior, defined by Trimpop (1994, p.9) as “any consciously, or non-consciously controlled behavior with a perceived uncertainty about its outcome, and/or about its possible benefits, or costs for the physical, economic or psycho-social well-being of oneself or others.” Risky behaviors within workplaces may turn into unsafe behaviors when employees undertake shortcuts and deviate from safety rules, thus enacting specific patterns of risky actions that jeopardize their own (and the collective) physical safety (Ghezzi et al., 2020). Noteworthy, risk-taking behaviors are not the simple product of individual initiatives and decisions but also depend on organisational determinants that can contribute to increasing (or decreasing) the likelihood of employee safety-oriented behavior, such as organizational culture norms and values (Petitta et al., 2017a, b). Most occupational accidents are attributed to behaviors that, at the time they are performed, are not perceived as potentially dangerous enough to create a severe accident (since they do not have the capacity to trigger a visible and immediate adverse effect), but whose existence can trigger hazardous situations and catastrophes (Martínez-Córcoles et al., 2018). According to the safety literature (Fleming & Lardner, 2002; Hollnagel, 1993; Mearns et al., 2001), risky behaviors encompass small deviations and circumventions (i.e., workarounds, shortcuts). An example of a deviation or circumvention can be illustrated by the maintenance operator who does not complete the four steps in the S.T.A.R. (Stop-Think-Act-Review) technique while checking a valve in a nuclear power plant. Skipping the last step may not appear to pose a risk to someone with years of experience in maintaining those same valves. Unlike non-compliance or violations, risky behavior also refers to acts outside the procedural scope perceived by the agent as safe and stable, but with the potential to incubate failures (Martínez-Córcoles & Stephanou, 2017). Indeed, the fact that an agent complies with rules and procedures s/he considers crucial for safety does not necessarily mean that s/he is not going to act in a risky way, quite the opposite: It is precisely because moving within the safety limits (relying on procedures) often creates a false feeling of safety, preventing workers from seeing the real impact of each of their actions.

It is worth emphasizing that mindful organizing proposes a counterintuitive approach to risks. Specifically, effective organizations are high reliability organizations but also high-risk organizations that take a variety of extraordinary steps in pursuit of error-free performance (Weick, 1987). For example, precautions might be wise in terms of safety but to the extent that they are designed to fit a simplified view of the world, they should be avoided by organizations which instead, encourage continuous attention and revision of how the system is operating. Effective organizations tend to develop anticipation skills and to predict and prevent a potential danger before damage is done but also cope with surprises in the moment. Therefore, they pay attention both to error-prevention and to error-containment. Conversely, traditional organizations tend to focus their effectiveness on anticipation of expected surprises and, more importantly, risk aversion by eliminating or delaying exposure to risks, and planned defenses against foreseeable risks (Amalberti, 2013). Rather than by risk aversion, effective organizations identify and avoid sets of outcomes they continually work to never experience, thus necessitating much more mindfulness, capability and alertness. As such, they need cognitive mechanisms that encourage the sensing of details but also an *un*integrated complexity in order to preserve its detailed variety, thus running the risk of appearing disorderly and unsafe. However, this apparent mess is aimed at promoting the requisite variety in order to anticipate errors and problems rather than undermining variety (Weick et al., 1999). Indeed, meta-analytic findings suggest that risk-taking behavior in organizations is associated with an increased chance of sustaining an injury except in the case of high skilled, risk-taking sports where the effect may be in the other direction (Turner et al., 2004).

We note that the ongoing mutual re-adjustment to situations (Landau & Chisholm, 1995) proposed by mindful organizing is consistent with the agentic approach to the functioning of the individual suggested by social cognitive theory (Bandura, 1986). According to reciprocal triadic determinism (Bandura, 1986), a person's behavior both influences and is influenced by personal factors as well as the physical and social environment. As such, an individual's behavior may be conditioned through the use of its consequences that human mind can process and elaborate in order to develop future courses of actions based on past experience. However, it is also possible to plan future behavior based on the mind’s ability to simulate multiple scenarios and anticipate potential consequences. Mindful organizing similarly suggests an agentic view of the individual constantly varying the routines or re-structuring procedures to fluctuating conditions and events while systematically engaging in processes of cognition that ensure alertness to seemingly insignificant weak signals and blind spots, and the envisioning of a nuanced picture of ongoing operations. In turn, this helps people to quickly detect if the system is acting in unexpected ways and thus, be prepared to handle unanticipated events or correct unintended consequences (Weick et al., 1999).

## Linking organizational culture, mindful organizing and organizational outcomes: A conceptual model

In this section we propose a conceptual model linking organizational culture, mindful organizing and organizational outcomes related to effective safety and crisis management, namely the Culture and Mindful Organizing model (CMO). First, we address the theoretical arguments that underpin the development of the proposed nomological network. Next, we build on the Attraction-Selection-Attrition theory as a matching principle of mindful organizing with organizational culture.

Each one of the five mindful organizing dimensions constitutes specific forms of shared thinking and acting among members, rooted in common mindsets that could be purposely cultivated by organizations through socialization (Weick et al., 1999). For example, preoccupation with failure entails a shared recognition of the complexity and interdependency of systems and their potential for surprises (thinking), and shared behaviors towards the enhancement of reporting failures (acting). Reluctance to simplify entails a shared concern to pay attention to the “overlooked” (thinking), and to bring together divergent perspectives that provide a greater ability to detect anomalies (acting). Sensitivity to operations implies the necessary creation of a collective mind that allows the integration of tightly-coupled interactive complexity as a dynamic operational process (thinking), and a constant search for sharing information and make interpretations collectively (acting). Commitment to resilience entails acknowledging human’s fallibility, murky technology and narrow specialties, and inevitable surprises (thinking), and embracing improvisation (acting). Lastly, deference to expertise relies on the shared belief that the necessary capabilities lie somewhere in the system, and on the shared thought of “looking for experts and not for bosses” when there is a need for quick decision making (thinking), and migration of decisions to the experts by formal delegation of power (acting). These collective world-views and mindsets shared among organizational members are important elements of organizational culture (Schein, 1985), understood as a set of beliefs and mental representations that contribute to shape and orient common ways of perceiving and behaving, thus underpinning the five processes of mindful organizing. Similarly, organizational culture in the area of safety represents a set of shared frames of reference that guide organizational members’ perception and interpretation of hazards, and that motivate and legitimize specific practices. Frames of reference are a prerequisite of detecting hazards, as they allocate attention, sensitize members to signals of danger, and provide conceptualizations of hazards (Nævestad, 2010).

**Fig. 2:**
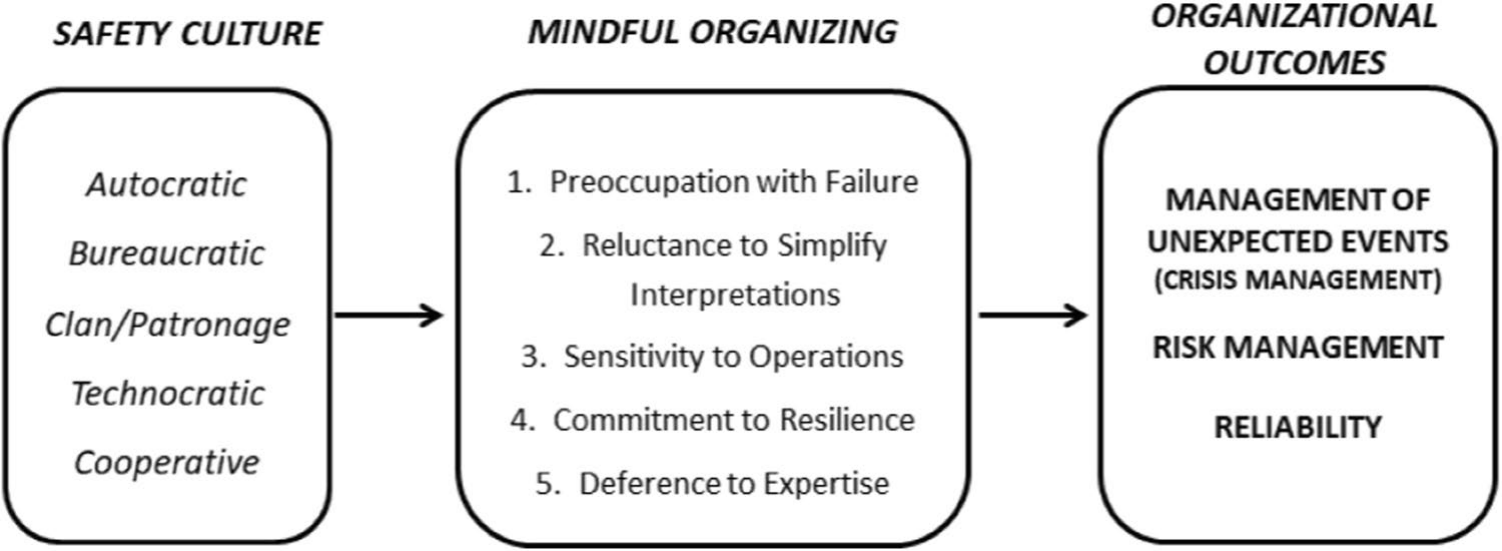
Conceptual model of organizational culture, mindful organizing and organizational outcomes (Culture and Mindful Organizing model)

Overall, organizational culture (e.g., safety culture) represents a contextual factor that qualifies as an antecedent of mindful organizing processes. In turn, mindful organizing boosts the development of the organization’s ability to manage unexpected events, thus laying the basis to predict later organizational effectiveness across multiple outcomes. As previously noted, the organization’s ability to handle uncertainties and the unexpected underpins its effective coping with disruptive events or rather, its crisis management skills. Moreover, continuous attention and alertness on details entailed by mindful organizing encourages effective risk management in terms of continuous monitoring and revision of how the system is operating in order to take steps to pursuit error-free performance. Related to the latter, mindful organizing processes also foster organization thinking and adaptive readjustments to situations thus underpinning the organization’s capacity to produce high quality collective outcomes on an ongoing basis or rather, system reliability. Figure 2 summarizes the proposed links among organizational culture, mindful organizing and organizational outcomes, and presents an overview of our overarching conceptual model (CMO).

Moving forward, organizational culture orients members’ actions and mindset and qualifies as an antecedent of mindful organizing processes, thus shaping the extent to which the specific features of each of the five processes can thrive or, conversely, suffocate in a given organization. A theoretical framework that potentially stands as a matching principle of mindful organizing requirements includes on the one hand, the cultural identity of the organization and on the other hand, the Attraction-Selection-Attrition (A-S-A) theory proposed by Schneider (1987). Specifically, the A-S-A model aims at understanding the etiology of organizational behavior and proposes that organizations are functions of the kinds of people they contain, thus contributing to explain the causes of processes (e.g., mindful organizing), structures and the technology of organizations. The A-S-A theory proposes three main propositions (Schneider, 1987). First, individuals are *attracted* to organizations whose members are similar to themselves in terms not only of personality but also values and beliefs that tend to match those of the collectivity of any given context (i.e., organizational culture). This first step of the A-S-A model is in line with Enriquez’s (1970) parameter “basic needs satisfied” used to define each culture type, and suggests that members’ cultural identity is also rooted in a dynamic of mutual satisfaction of the needs that members hold and that the organization, defined by its cultural values, is able to satisfy. Second, organizations are more likely to *select* and include in their context those who possess knowledge and skills, as well as values and beliefs, similar to the ones their existing members possess. Third, over time, members who do not fit in well are more likely to experience, or generate, *attrition* and thus leave. This latter point is most important in order to understand the concept that members whose actions are *not* in line with the organizational culture tend not to be integrated. Overall, the three A-S-A forces are part of a cycle that restricts the range of types of people in an organization, and suggests that the personal characteristics of those who work for an organization are likely to become more similar and homogeneous over time. This also explains why cultural features tend to remain consistent and perpetuate inside the context, thus leading to the consolidation of organizational culture.

**Fig. 3:**
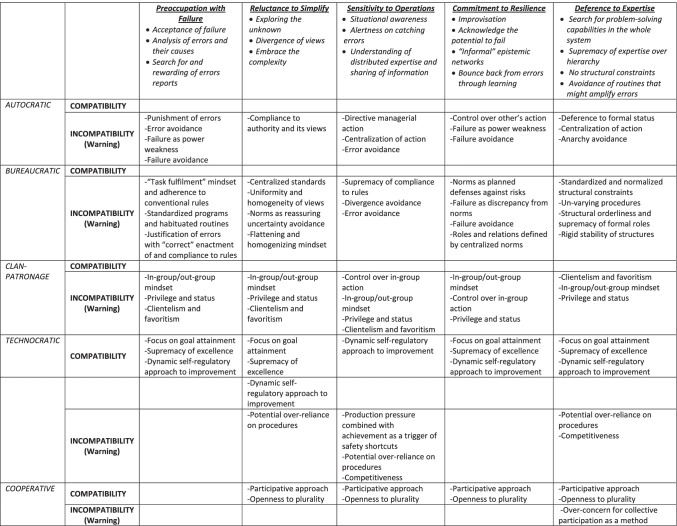
Assessing the compatibility between organizational culture and mindful organizing

Building on the A-S-A theory (Schneider, 1987) and Enriquez’s (1970) cultural typology, we propose a conceptual framework aimed at assessing the extent to which each culture type is compatible versus incompatible with the features of each mindful organizing process (i.e., preoccupation with failure, reluctance to simplify interpretations, sensitivity to operations, commitment to resilience and deference to expertise). Figure 3 summarizes the overall approach and reports the arguments for assessing an organization’s cultural compatibility, or conversely, incompatibility with mindful organizing processes. For instance, in an *autocratic safety* culture, the leaders exert an autocratic action, subordination is expected and rewarded; compliance to authority and its views is rewarded; failure and errors are considered as power weakness and therefore avoided; identification with authority favours imitation of behaviors and deference to authority and formal status, thus avoiding anarchy. All of these cultural elements stand as incompatible with acceptance of failure and analysis of errors essential to the preoccupation with failure, divergence of views required by the reluctance to simplify, distributed expertise that underpins sensitivity to operations, acknowledgement of the potential to fail and “informal” epistemic networks required by the commitment to resilience, and the supremacy of expertise over hierarchy and the lack of structural constraints essential for deference to expertise.

In *bureaucratic* cultures, a “task fulfilment” mindset and an adherence to conventional rules that are planned defenses against risks and reassuring standpoints that help avoid or reduce uncertainty is prevalent (Grote, 2004, 2007). Failure is conceived as discrepancy from norms and thus avoided; errors tend to be justified with “correct” enactment of and compliance to rules; there is homogeneity of views and structural orderliness that lead to standardized programs and habituated routines. All of these cultural features are incompatible with the search for errors as expected by preoccupation with failure, the exploration of the unknown and acceptance of uncertainty required by reluctance to simplify, the mapping of distributed and differential expertise necessary for sensitivity to operations, the improvisation and informal networks required by commitment to resilience, and the loose structural constraints and avoidance of routines indispensable for deference to expertise.

Within the *clan-patronage* culture, the instrumentality of group membership is fundamental. Groups enact reciprocally beneficial exchanges between members and people with power. The paternalistic or patronage component is manifested by the superiority and control of those in power who protect group members in search of security. The in-group/out-group mindset is combined with privilege and status, clientelism and favoritism. Individuals in power have control over in-group action. The model of behavior is two-face: informal with in-group members and formal with externals. Consistently, all of these cultural characteristics are incompatible with the acceptance of failure and rewarding of errors required by preoccupation with failure, the divergence of views required by reluctance to simplify, the distributed expertise and sharing of information (e.g., with out-group) that underpins sensitivity to operations, the acknowledgement of the potential to fail and “informal” epistemic networks required by commitment to resilience, and the search for problem solving capabilities in the whole system (e.g., in the out-group), as well as the supremacy of expertise over hierarchy essential for deference to expertise.

The *technocratic* culture is characteristic of contexts which are result oriented, as well as focused on competence, innovation, continuous self-improvement seeking and competition. As such, some cultural features qualify as compatible and in line with mindful organizing processes whereas others may stand as incompatible. Specifically, the focus on goal attainment, the supremacy of excellence and a dynamic self-regulatory approach to individual and system improvement may be compatible with the search, analysis and rewarding of errors required by preoccupation with failure and the divergence of views as well as openness to the unknown related to reluctance to simplify. The dynamic self-regulatory approach to constant improvement seeking is also compatible with alertness to situations and errors and the sharing of information required by sensitivity to operations. The focus on attainment, excellence and self-regulation also matches improvisation and bouncing back from errors through learning that underpins commitment to resilience, as well as the supremacy of expertise over hierarchy and problem-solving orientation required by deference to expertise. Conversely, when cultural traits of over-reliance on procedures per se and competitiveness exist, then problems may rise in the flexibility, sharing of information and alertness of errors essential for reluctance to simplify, sensitivity to operations and deference to expertise.

Finally, within a *cooperative* culture**,** value is given to the achievement of consensus and the participation of all members during decision making. It is not to be misunderstood with spontaneous cooperation among people. Conversely, it implies widespread rules that require the contributions of all individuals to collective outcomes due to the organizational assumption that the resultant whole is greater than the sum of its parts. As such, the participative approach and openness to plurality are compatible with the divergence of views required by reluctance to simplify, the understanding of distributed justice and sharing of information related to sensitivity to operations, the informal epistemic networks needed by commitment to resilience, and the loosening of hierarchy essential for deference to expertise. However, the cooperative concern for participation as a method may stand as scarcely compatible with the lack of structural constraints required by deference to expertise and velocity of action required by all mindful organizing processes.

Overall, the proposed overarching conceptual framework combined with the A-S-A approach provides organizations with an operational tool that allows them to a) audit their culture features through culture type diagnosing, b) identify where they stand in terms of the culture features they possess in comparison to those that are more suitable for mindful organizing processes, and c) design the necessary culture change steps and take action in order to actively disseminate new cultural information at several ascending levels, ranging from artifacts that symbolize a corporate personality (the easiest to change), to shared values, up to the highest level of abstraction of shared assumptions which are also the hardest to change (Weick & Sutcliffe, 2007).

## Discussion

According to the World Health Organization (2021), the Covid-19 pandemic has generated nearly 180 million of confirmed cases and 4 million deaths world-wide. As for the number of deaths in occupational settings due to work-related Covid infection, while there is a scarcity of statistics on Covid-related injuries because government Agencies might rarely track workplace exposure to the virus (Johnston, 2021), some initial reports (e.g., North Carolina Department of Labor, 2021) suggest that Covid-19 fatal injuries could reach up to thirty percent of all workplace deaths in some countries. Moreover, the International Labor Organization (2021) reports that workers around the world annually experience nearly 340 million occupational accidents and the updates indicate an increase of accidents and illnesses despite the work stoppages due to lockdowns. Not only, the pandemic has faced all nations world-wide with the unprecedented challenges of controlling the spread of infection and managing a surprising and complex medical crisis, as well as caused organizational settings to effectively counteract an unchartered and rapidly escalating medical emergency while needing to preserve the safety and well-being of workers. Ultimately, safety and crisis management have become increasingly intertwined in order to successfully cope with the currently unstable and complex environment and its disruptive unexpected events. The geopolitical turmoil caused by the consequences of the war in Ukraine is just an additional striking challenge for organizational efficacy and crisis management in dealing with unforeseeable emergencies. Organizations need to organize mindfully in order to operate in volatile, uncertain and complex environments while simultaneously adaptively learning and reliably performing in order to survive (Vogus, 2011). Noteworthy, cultural differences in the mindset of people have emerged as a key factor in effectively counteracting and controlling Covid-19, within both nations and organizations (Erman & Medeiros, 2021).

The purpose of the current study was to propose the Culture and Mindful Organizing (CMO) model as an integrative framework linking organizational culture, mindful organizing and organizational outcomes (i.e., crisis management, risk management, reliability), and delineate arguments to address the match/mismatch between mindful organizing and culture types. By using the A-S-A framework (Schneider, 1987) as a matching principle and systematically comparing the specific features of mindful organizing processes with idiosyncratic aspects of different organizational culture types from Enriquez’s (1970) typology, our study demonstrates that organizational culture determines the way an organization develops mindful organizing and its subsequent ability to handle unexpected and disruptive events that likely jeopardize organizational effectiveness and safety. Moreover, it provides a methodology to intervene on the cultural underpinnings of mindful organizing.

### Theoretical implications

Our study has implications for the extant literature in the areas of mindful organizing, organizational culture, crisis management and safety. First, our CMO model responds to the recent call from researchers in the field of safety and mindful organizing (e.g., Martínez-Córcoles & Vogus, 2020) to examine organizational contextual variables as causes of mindful organizing as well as to address the still unanswered questions on how to enhance mindful organizing processes (Klockner, 2018). While both safety culture and mindful organizing are acknowledged as two important factors in managing the unexpected, the link between the two phenomena has not been theoretically developed. Our study bridges the still disparate fields of organizational safety culture and mindful organizing. Indeed, the literature suggests that mindfulness is a more social construct than its name implies (Sutcliffe et al., 2016) and posits a link between organizational culture and mindful organizing (Klockner, 2018; Ray et al., 2011; Vogus & Sutcliffe, 2012). However, the research in this domain mainly focuses on the role of leaders in developing a culture of mindfulness and argues that organizational mindfulness is evident when leaders create cultures that encourage rich thinking and a capacity for action (Ray et al., 2011). While those contributions speculate on the indirect effect of organizational culture on mindful organizing through leadership, the current paper specifically theorizes on how organizational culture may directly affect mindful organizing processes. Noteworthy, we extended earlier research showing a direct relationship between organizational culture and mindful organizing. In particular, the use of the ASA framework (Schneider, 1987) as a conceptual foundation allowed us to disentangle the way specific culture features of a given organization may fuel, or conversely constrain, each one of the mindful organizing processes. As such, our CMO model enlarges the scarce existing knowledge on the importance of cultural assumptions and values in enhancing mindful organizing processes (e.g., McDonald et al., 2019).

Second, research is underdeveloped regarding where and how mindful organizing arises in work settings and its associated consequences (Sutcliffe et al., 2016). Moreover, theorizing on mindful organizing has often been critiqued for being too narrow in its conceptualization, too micro in its level of analysis and inadequately socially embedded (Martínez-Córcoles & Vogus, 2020). Our study informs the organization theory and safety literature by demonstrating a new additional explanatory mechanism for the relationship between culture and organizational outcomes (e.g., risk management, organizational reliability). By proposing mindful organizing as a mediator of the link between organizational culture and organizational outcomes, the current study moves forward the traditional view of mindful organizing as the main source for effective safety and crisis management and undertakes a more holistic and nuanced conceptualization of the construct and its relationships with other relevant phenomena in organizations. Specifically, the suggested view of mindful organizing as an “in-between” phenomenon opens a new research avenue, which refers to the unexplored area of the contextual dynamics affecting how mindful organizing arises in work settings and its associated consequences (Sutcliffe et al., 2016). The integrative framework proposed here facilitates theoretical underpinnings.

Finally, our study adds to the organizational culture literature by expanding its causal link with organizational processes and managing techniques as well as effective organizational performance. Specifically, we fill the gap of existing knowledge on how organizational culture contributes to an adaptive performance in uncertain, volatile and complex environments. Indeed, organizational culture tends to stabilize the organization (i.e., aligning expectations, orienting and driving members towards concrete accepted routines and behaviors; Schein, 1985). As such, one may argue that this stabilizing effect of culture may prevent members from acting dynamically and thinking, interpreting and behaving mindfully (e.g., projecting new courses of action beyond the traditional ones to solve unexpected problems). Noteworthy, the cultural typology (Enriquez, 1970) and A-S-A framework (Schneider, 1987) proposed in the current study enables the assessment of which basic assumptions and values relate to mindfulness capability, thus demonstrating that specific organizational culture types embed features that have the potential to enhance the organization’s capability to operate effectively and safely.

### Practical implications

Our study has also several implications for practice. While the mindful organizing framework has been initially proposed in association to high reliability organizations (e.g., nuclear plants, aviation), recent developments suggest its applicability to all types of organizations willing to improve their safety (Martínez-Córcoles & Vogus, 2020). This has become increasingly evident during the Covid-19 outbreak that has unfolded the necessity of both anticipation and resilience skills (i.e., mindful organizing dimensions) to successfully counteract the medical crisis. At the very beginning of the pandemic in March 2020, Pisano et al. (2020) discussed lessons from Italy’s response to the Coronavirus as the biggest crisis since World War II. The authors remarked on the difficulties of making decisions in real time, when a crisis is unfolding and how the systematic failure to act upon existing information was related to leaders’ difficulty in figuring out how to act in highly complex situations. Moreover, they suggested that an effective response to the virus required the orchestration of a coherent system of actions taken simultaneously and therefore a shift from patient-centered models of care to a community-system approach. More importantly, they stressed the relevance to learn from both successes and failures and the willingness to change actions accordingly, thus emphasizing the critical role of learning from past and current experiences, especially at the time of heightened uncertainty. On a different, yet related note, extensive literature on sustainability of organizational processes under uncertain and unstable conditions (e.g., the Covid-19 outbreak) suggests that reliable organizational outcomes such as sustainable economic performance and innovative work behavior were determined by empowering leadership in resilient organizations (Faulks et al., 2021). Similarly, enterpreneurial leadership style proved to be an effective strategy for managing uncertainty and ensure sustainable economic performance, or even thrive, across different industry sectors (Alsharif et al., 2021).

Overall, an effective response to the medical crisis would require the adoption of a mindset (as well as systems and processes) that facilitates a decision-making approach that is systemic, prioritizes learning, and is able to quickly scale successful experiments and identify and shut down ineffective ones. Interestingly, the suggested crisis management approach seems to call into action all mindful organizing processes, from attention to failure and system involvement at all levels up to deference to expertise and facing uncertainty through continuous learning. Moreover, it concludes on the relevance of “adopting a mindset” (i.e., culture) that may facilitate all these processes, thus demonstrating the practical applicability of mindful organizing to crisis management in all business fields, not only safety, including medical and institutional (e.g., political) sectors.

As such, organizations willing to implement mindful organizing as a work and crisis management practice are warned of the unavoidable prerequisite of a compatible cultural mindset within their context. Our proposed conceptual model (CMO) and related matching methodology between culture type and mindful organizing is a useful tool here. Specifically, organizations might profile their cultural identity and define a map of matching areas as well as gaps with mindful organizing in order to undertake interventions that enable the entire system to adjust accordingly. As a foundation, intervention programs might fruitfully aim to enhance management and employees’ awareness of the shared behavioral patterns among members and their influence on the qualities of their perception and thinking processes. To this end, research suggests that in order to modify existing cultural patterns, interventions should focus on changing programs that help disseminate and crystallize new policies and norms as well as new organizational beliefs and behaviors in line with mindful organizing (DeJoy, 2005). Moreover, organizations might provide employees with individual tools (e.g., mindfulness practices, reflective activities) to train the mind to control attention and actions (Weick & Putnam, 2006).

As noted, the national culture of any country world-wide shapes the practices of their fight against Covid-19 and their level of success in managing the crisis (Gokmen et al., 2021; Han et al., 2020). Specifically, using Hofstede’s (1991) conceptualization of culture, defined as the collective programming of the mind that distinguishes the members of a group or category of people, research suggests that power distance negatively affects the rate of total COVID-19 cases in the nation while individualism positively impact the spread rates. Moreover, across different countries, power distance and individualism have effects on the success of a nation in controlling the COVID-19 pandemic. Multinational organizations willing to assess how different local cultures affect mindful practices in their national branches, despite the uniform motherhouse guidelines, would greatly benefit from our conceptual model (CMO) and related comparing methodology. The technique proposed in our study can be adapted for these purposes. Specifically, our study builds on the A-S-A theory (Schneider, 1987) as a matching principle to assess the compatibility of mindful organizing with organizational culture, and draws upon Enriquez’s (1970) culture typology as a diagnostic model. While Enriquez’s typology was proposed because it provides detailed features of each culture type and therefore, allows a fine-grained assessment of the organizational identity, Hofstede’s (1985) culture model could be used as an alternative theoretical foundation to facilitate cross-country comparison.

### Strengths, limitations and future perspectives

While this study makes several contributions to the extant literature, it also suffers from some limitations that should be addressed in future research efforts. First, our study is an important first step at demonstrating the organizational culture underpinnings of mindful organizing; yet, the proposed nomological network of the CMO model should be empirically tested on data collected in organizations from different occupational settings and across different national contexts in order to ensure ecological validity. Specifically, future studies might include organizational-level mindfulness scales such as the short 9-item Mindfulness Organizing Scale from Vogus and Sutcliffe (2007b) or the more extensive 48-item Mindful Organizing assessment tool from Weick and Sutcliffe (2007), along with individual-level crisis and risk management scales (e.g., Veil et al., 2008). Moreover, given the multilevel nature of our proposed conceptual model including organizational- (i.e., culture, mindful organizing) and individual-level variables (e.g., crisis management coping), future research efforts should target people nested within a minimum of 50 to 100 organizations to obtain hierarchical data and reliable multilevel structural equation modelling results (Heck & Thomas, 2020; Hox et al., 2017).

Second, Enriquez’s (1970) cultural typology allows a fine-grained diagnosis of the prototypical features of an organizational identity; yet, multimethod assessments of organizational culture integrating the proposed quantitative approach with a qualitative investigation of more idiosyncratic and nuanced aspects of the context may help in better defining its compatibility with mindful organizing characteristics. Finally, the model of mindful organizing proposed by Weick et al. (1999) is cognition-centered and ascribes a crucial role to individual and social processes of cognition. However, recent literature suggests that overwhelming emotions may rise especially in the heat of the moment in dealing with complexities and failures (Oliver et al., 2019) and therefore, attention should be given to how humans deal with hazards and/or errors under pressure (Woods & Hollnagel, 2006). Moreover, in line with Vogus (2011) suggesting that weak signals are often expressed emotionally through subtle changes in non-verbal signaling such as tone, facial expression, and body language, we note that emotion-related factors are inherently involved in all mindful organizing processes, such as preoccupation (i.e., a feeling), alertness (i.e., a generalized sense of activation associated to feelings of anxiety) and dealing with uncertainty and unexpected events (i.e., an anxiety-related process). As such, the proposed framework of mindful organizing could be further expanded and include the role of context-related emotional dynamics (e.g., emotional contagion; Petitta & Naughton, 2015) in order to explore how general cognitive skills and capabilities to anticipate and solve problems are intertwined with emotion management skills when dealing with complexity under pressure, high-hazard contexts and the avoidance of dysfunctional and dangerous actions. Hopefully, the conceptual model proposed here will shed light on the link between organizational culture, mindful organizing and safety, and provide the basis for future theory development as well as new orientations for practitioners.

## Data Availability

The current study has no data associated.
